# Adverse Events in Symptom Management Interventions Using Immersive Virtual Reality in Older Adults With Cancer

**DOI:** 10.1093/geroni/igaf122.3199

**Published:** 2025-12-31

**Authors:** Kailei Yan, Victoria Loerzel

**Affiliations:** University of Central Florida, Orlando, Florida, United States; University of Central Florida, Orlando, Florida, United States

## Abstract

Emerging evidence shows that Virtual reality (VR) based interventions can improve motivation and engagement in health-promoting behaviors, offering a promising approach for cancer patients who struggle with symptom burden during and after treatment. While VR-based interventions show therapeutic potential, its effectiveness cannot be fully validated without a critical evaluation of its potential risks. The goal of this report is to describe the adverse events (AE’s) reported from our systematic review of randomized controlled trials (RCTs) of symptom management interventions using VR among cancer populations, especially older adults. Among the 29 studies reviewed, 2 studies included adults over age 65. Of these, 11 did not present information on AE’s and 5 stated that no AE’s were observed. In the remaining studies, difficulty concentrating, mild nausea, tired or aching eyes, moderate fatigue, drowsiness, dizziness and vomiting were reported. Of the 2 studies with older adults, one reported similar AE’s as above, the other recorded no AE. One study found that pre-existing symptoms before VR interventions were significantly associated with post-VR AE’s. Validated instruments were used to assess AE’s in only 5 studies. One study used semi-structured interviews. Overall, this review identified substantial gaps in the reporting of adverse events, especially among older adults. This emphasizes the need for more rigorous documentation and evaluation of the risks associated with VR interventions. Because, current clinical trial reporting primarily focuses on severe AE’s, such as impairment on overall health, a more VR specific approach of evaluation of AE’s, including vestibular and psychological impacts, is needed.

